# Sub-chronic treatment with high doses of ascorbic acid reduces lead levels in hen eggs intentionally exposed to a concentrated source of lead: a pilot study

**DOI:** 10.1186/s40360-020-0389-4

**Published:** 2020-03-02

**Authors:** Ramzi Shawahna, Ahed Zyoud, Elaf Haj Yahia, Rahma Sulieman, Abeer Haddad, Mohammad Makhlof, Bilal Abu-Hilal, Ghulam Murtaza, Hikmat Hilal

**Affiliations:** 1 Department of Physiology, Pharmacology and Toxicology, Faculty of Medicine and Health Sciences, An-Najah National University, New Campus, Building: 19, Office: 1340, P.O. Box 7, Nablus, Palestine; 2 An-Najah BioSciences Unit, Centre for Poisons Control, Chemical and Biological Analyses, An-Najah National University, Nablus, Palestine; 3 Department of Chemistry, Faculty of Science, An-Najah National University, Nablus, Palestine; 4 Department of Medicine, Faculty of Medicine and Health Sciences, An-Najah National University, Nablus, Palestine; 5 Faculty of Agriculture and Veterinary Medicine, An-Najah National University, Nablus, Palestine; 6 Department of Pharmacy, COMSATS University Islamabad, Lahore, Pakistan

**Keywords:** Ascorbic acid, Ecotoxicology, Eggs, Heavy metals, Lead poisoning, Toxicity

## Abstract

**Background:**

Hen eggs contaminated with lead can be harmful to the health of children and adults. The objective of this pilot study was to investigate if sub-chronic treatment with ascorbic acid can reduce lead levels in the different parts of hen eggs after intentionally exposing the laying hens to a concentrated source of lead.

**Methods:**

Clinically normal mixed-breed egg laying hens (*n = 18*) were used in this pilot study. Hens were exposed to a concentrated source of lead (200 mg/kg_body weight_/day lead acetate) for 1 week. Subsequently, egg laying hens were either treated with sub-chronic doses of ascorbic acid (500 mg/kg_body weight_/day) or left untreated for 4 weeks. Lead levels were assessed in egg-shell, egg-albumen, and egg-yolk samples using a graphite furnace atomic absorption spectrophotometer.

**Results:**

Lead levels increased significantly (*p*-value < 0.01) from baseline in egg-yolk, egg-albumen, and egg-shell samples following 1 week exposure to lead acetate. Sub-chronic treatment of egg laying hens with high doses of ascorbic acid could bring statistically significant reduction (*p*-value < 0.01) in lead levels in egg-yolk, egg-albumen, and egg-shell samples after intentional exposure to a concentrated source of lead.

**Conclusions:**

Findings of this pilot study showed that sub-chronic treatment of egg laying hens with ascorbic acid can reduce lead levels in different egg parts after intentional exposure to a concentrated source of lead. Supplementing feedstuffs and water with sources of ascorbic acid could be beneficial in reducing lead levels in hen egg tissues following environmental exposure. Further studies are still required to investigate if ascorbic acid can reduce lead levels in other chicken tissues.

## Background

Hen eggs possess high nutritional properties that made them one of the major sources of proteins and other nutritional elements in the diet of many nations [1–3]. As eggs are known to contain essential elements for the normal growth and development of the human body, hen eggs are often added to enhance the nutritional value of a variety of meals and plates [4]. Children as well as adults around the globe consume large quantities of hen eggs on daily basis. A recent report by the Palestinian Central Bureau of Statistics indicated that, on average, a Palestinian household consumed around 4 kg of eggs per month [5]. Generally, hen eggs are widely used because they are familiar, economic, and available in large quantities [4].

It has been suggested that hens can serve as biological filters and eggs laid in environments free from or low with contaminants are safe for consumption. However, a growing body of research has shown that eggs laid by hens exposed to environmental pollution contain considerable levels of potentially harmful elements such as heavy metals [6, 7]. It has been suggested that after ingestion by hens, heavy metals can deposit in different tissues including eggs laid by hens exposed to pollution [2, 8]. Therefore, assessing foods for potentially harmful toxic metals has been recently recognized as an important health issue because exposure to heavy metals can be harmful to the health of children and adults [9, 10].

Lead is one of the most ubiquitously available potentially harmful heavy metal in our environment [11, 12]. Today, many sources of lead contamination were identified. These sources include water, air, and soil [6, 12, 13]. Increasing exposure to environmental lead has been attributed to recent industrialization and urbanization in some regions [13, 14]. Recent studies have shown that plants and grains grown upon soils contaminated with lead contained elevated levels of this toxic heavy metal [10, 15, 16]. Therefore, it would not be surprising that birds and animals grazing on contaminated grains would be found to have elevated levels of this toxic heavy metal [6, 17]. Lead would probably be deposited in various tissues including eggs laid by birds exposed to environmental lead [18, 19]. Ingestion of lead contaminated hen eggs can increase lead levels in children and adults [10]. A recent study conducted on breastfeeding women from the West Bank of Palestine showed that about 19% of the breast milk samples analyzed contained lead levels above those recommended by the World Health Organization (WHO) [14]. Studies have found a link between higher lead levels and hypertension, renal failure, and depression [2, 20, 21]. Other studies have also found significant associations between elevated lead levels and hematic, renal, gastrointestinal, mental, cognitive, and developmental impairments [22–24]. Moreover, children exposed to lead were also shown to report poor academic and scholastic achievements [22–24].

Reducing exposure to potentially toxic heavy metals is a health priority. Previous studies investigated the use of several chelators to decrease toxicity in the incidents of exposure to lead [25]. Some of the previously used chelators were potentially toxic and could not be easily removed from tissues, thus, could present another risk to health [13, 25]. In addition to its well-known antioxidant properties, ascorbic acid (vitamin C) is known to possess metal chelating activities [26]. The use of ascorbic acid has been suggested as a dietary strategy in the management of lead toxicity [27]. Ascorbic acid was shown to be beneficial in protecting cells and tissues from deleterious effects in the events of external stress [28]. A previous investigation showed that sub-chronic treatment with high dose of ascorbic acid was shown to decrease lead levels in the blood of broiler hens intentionally exposed to a high concentration source of lead [29].

Currently, little is known if sub-chronic doses of ascorbic acid could bring significant reduction in the levels of lead in hen egg portions after intentionally exposing hens to a concentrated source of lead. In this pilot study, we investigated the effects of sub-chronic doses of ascorbic acid on lead levels in the different portions of hen eggs after intentionally exposing the laying hens to a concentrated source of lead*.*

## Methods

### Ethical considerations and animal welfare

All procedures in this study were conducted in compliance with the ethical principles followed at An-Najah National University as well as the internationally recognized animal care and use regulations. The study protocol was approved by the research and ethics committee of An-Najah National University. The hens used were treated humanely and their vital signs were monitored and recorded throughout the different stages of the study. A licensed veterinarian observed the hens for signs of toxicity and ensured their welfare throughout the different stages of the study. Once the study was terminated, all hens were euthanized humanely in accordance with the guidelines of the European Commission, the Humane Society of the United States, and the American Veterinary Medical Association (AVMA). This study is reported in accordance with the Animal Research: Reporting of in vivo Experiments (ARRIVE) Checklist which is provided in Additional file 1.

### Study design

In this pilot study, we obtained clinically normal mixed-breed adult egg laying hens (*n = 18*) from local raisers in the West Bank of Palestine. The sample size was informed by previous studies after considering the number of eggs that could be laid by each hen every day [6, 30, 31]. Hens were housed separately in cages in the poultry housing facility of the Faculty of Agriculture and Veterinary Medicine, An-Najah National University. Once procured into the poultry housing facility, hens were given 1 week of acclimatization period during which hens had access to feedstuff and water ad libitum*.* Eggs laid during the acclimatization period were collected to assess baseline lead levels.

### Control and treatment groups

**Fig. 1:**
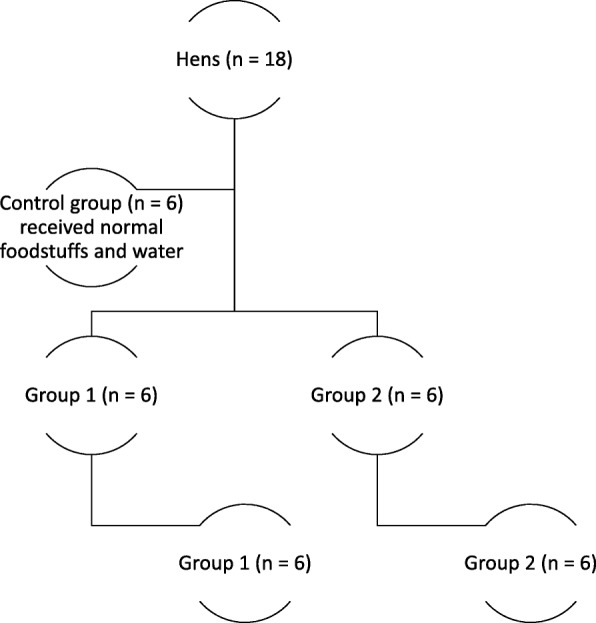
The different stages of the study

Following the acclimatization period, hens were divided randomly into groups each of 6 hens. A group of hens (*n = 6*) received normal feedstuff and water throughout the study. This group served as the control group (Fig. 1). The rest of hens were divided into 2 groups (G1 and G2). All hens in both groups received lead acetate (200 mg/kg_body weight_/day) mixed with small portions of feedstuff and water on daily basis for 1 week. The decision to use this dose was informed by previous studies on the neurobiological changes of lead intoxication in different species [32–35].

Investigators ensured that the portions mixed with lead acetate were consumed completely by each hen before giving free access to water and feedstuff and before the next day dose. Eggs laid during this 1 week period were collected, labelled, and stored separately in suitable plastic transparent bags at 4 °C until the time of preparation for analysis.

To investigate if a high dose daily supplementation with ascorbic acid can reduce elevated lead levels in the different portions of hen eggs, hens in G1 received ascorbic acid (500 mg/kg_body weight_/day) mixed with water and feedstuff on daily basis for 4 weeks. The decision to use this dose was informed by previous studies in which ascorbic acid was investigated in the management of lead intoxication [36, 37]. Eggs laid during this period were collected, labeled, and stored separately in suitable plastic transparent bags at 4 °C until the time of preparation for analysis.

To investigate if the reduction in elevated lead levels in the different portions of hen eggs resulted from ascorbic acid treatment and not from another endogenous detoxification pathway, hens in G2 did not receive any ascorbic acid supplementation during the following 4 weeks period. Similarly, eggs laid during this period were collected, labeled, and stored separately in suitable plastic transparent bags at 4 °C until the time of preparation for analysis. At the end of the study, all hens were euthanized using an overdose of pentobarbital (200 mg/kg i.v.).

### Preparation of hen eggs for analysis

Before analysis, all hen eggs collected were cleaned with soap, water, and soaked in 1% triton-X for 4 h to remove any external contamination [6]. Eggs were then rinsed in distilled water. All eggs were boiled in a water bath until solidification. Eggs were separated into eggshell, egg-albumen (white) and egg-yolk (yellow). Each egg was treated separately.

### Analytical procedure

To remove all water, egg parts were transferred to an oven and dried at 120 °C for 8 h. Aliquots of 1 g of each part were mixed with 1 mL of concentrated HNO_3_ into a crucible and ashed for 1 h at 540 °C. Ash aliquots were soaked with 10 mL (3:1) mixture (1 M HNO_3_ and 1 M HClO_4_) for 1 h. Aliquots were then filtered and the supernatants were analyzed for lead levels.

Determination of lead levels was done using graphite-furnace atomic absorption spectrophotometer (iCE™ 3500 Atomic Absorption Spectrometer, Thermo Scientific, UK). Determination of lead levels in fresh eggs using a graphite-furnace atomic absorption spectrophotometric method was previously described by Kiliç et al. [38]. The plastic bags into which the eggs were stored were soaked in distilled water overnight. Water samples were analyzed for lead levels to ensure that the plastic bags were not a source of lead contamination. All glassware and crucibles were incubated in 10% HNO_3_ for 24 h to hinder adsorption of lead onto the surfaces. Lead levels were computed against calibration curves established in similar matrices. Eggshell, egg-albumen (white), and egg-yolk (yellow) samples were analyzed on dry-weight basis. The chemicals used in this study were of analytical grade. The analytical method used had a limit of detection of 1.88 ng/g as previously described by Kiliç et al. [38].

### Statistical analysis

Data were entered into GraphPad Prism v.6.0 for Windows. Statistical analysis was performed using Analysis of Variance (ANOVA) with Bonferroni multiple comparisons. Statistical significance was considered as: * when the *p*-value was < 0.05 and ** when the *p*-value was < 0.01.

## Results

### Lead levels in egg yolk samples

**Fig. 2:**
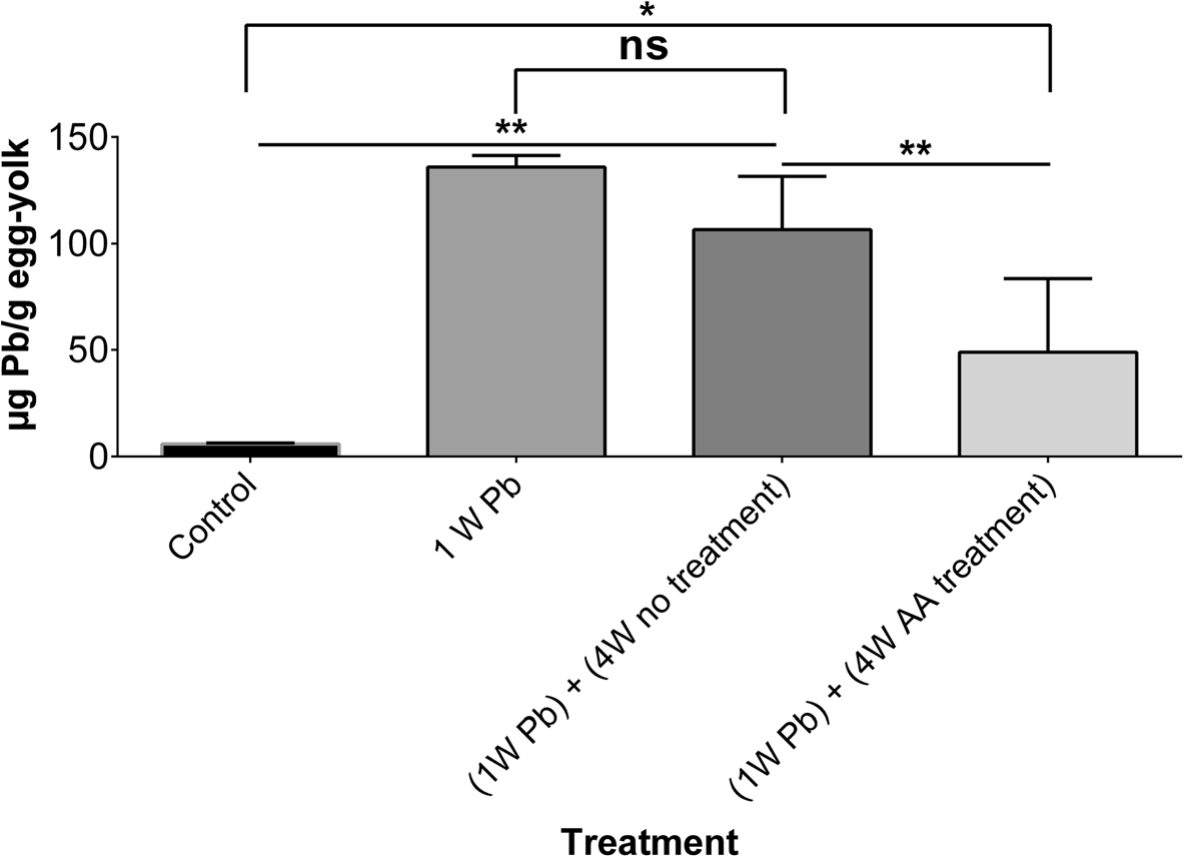
Lead (Pb) levels in egg-yolk samples at baseline (control), following exposure to lead acetate (200 mg/kg_body weight_/day) for 1 week (1W Pb), 3 weeks without (4W no treatment), and 3 weeks with (4W AA treatment) treatment with high daily doses (500mg/kg_body weight_/day) of ascorbic acid. A total of 6 samples were analyzed. ns: not statistically significant, **: *p*-value < 0.01, *: *p*-value < 0.05

Baseline lead levels were 5.9 ± 0.4 μg Pb/g egg-yolk. Lead levels in egg yolk increased significantly (*p*-value < 0.01) to reach 136 ± 5.4 μg Pb/g egg-yolk after intentionally exposing hens to lead acetate (200 mg/kg_body weight_/day for 1 week). These elevated lead levels did not decrease significantly (107 ± 25 μg Pb/g egg-yolk) after 4 weeks of no treatment (*p*-value > 0.05). Interestingly, lead levels in egg-yolk samples significantly decreased (48.9 ± 34.6 μg Pb/g egg yolk) following 4 weeks of treatment with sub-chronic doses of ascorbic acid (500 mg/kg_body weight_/day). Lead levels in egg-yolk samples are shown in Fig. 2.

### Lead levels in egg-albumen samples

**Fig. 3:**
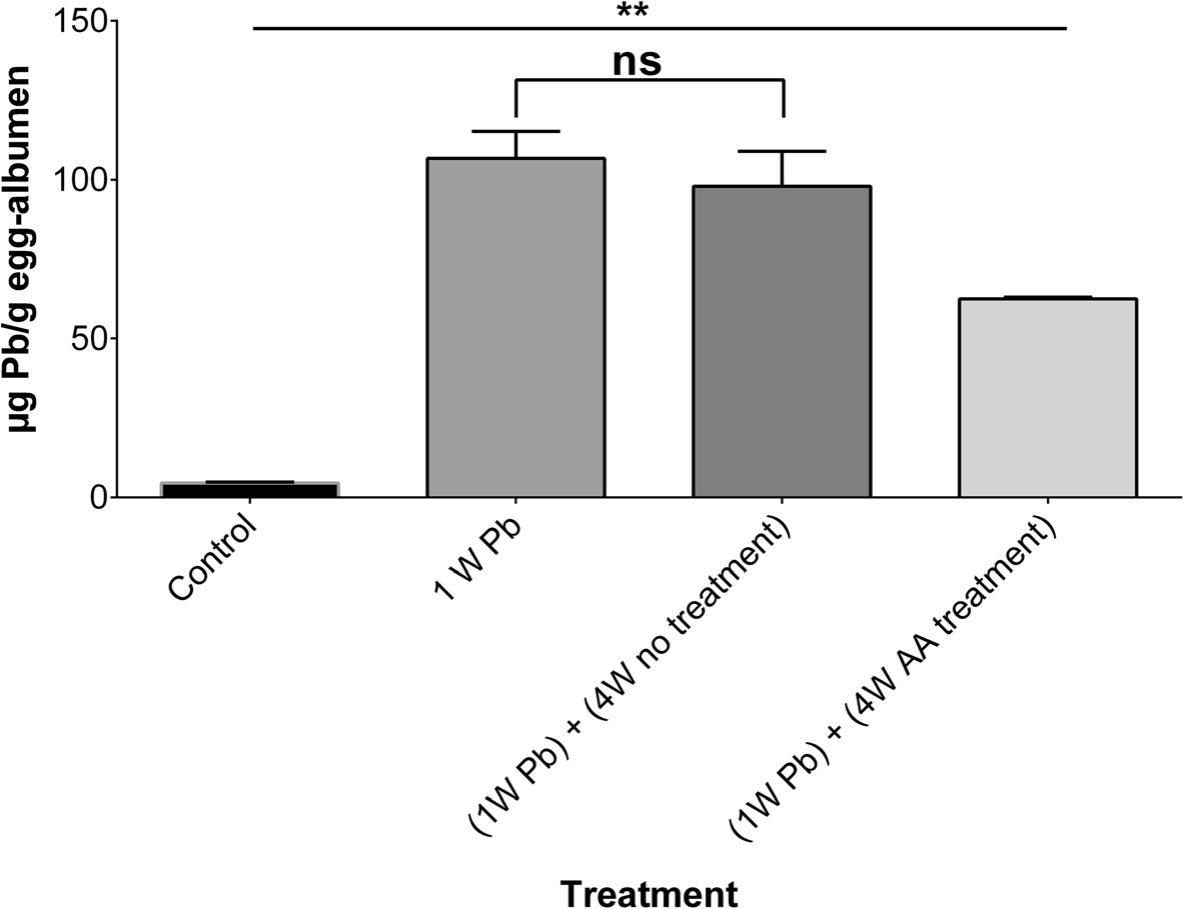
Lead (Pb) levels in egg-albumen samples at baseline (control), following exposure to lead acetate (200 mg/kg_body weight_/day) for 1 week (1 W Pb), 4 weeks without (4W no treatment), and 4 weeks with (4W AA treatment) treatment with high daily doses (500 mg/kg_body weight_/day) of ascorbic acid. A total of 6 samples were analyzed. ns: not statistically significant, **: *p*-value < 0.01

Baseline lead levels were 4.5 ± 0.3 μg Pb/g egg-albumen. Similar to egg-yolk, lead levels significantly (*p*-value < 0.01) increased in egg-albumen samples to 107 ± 8.5 μg Pb/g egg-albumen following 1 week of exposing hens to lead acetate. Lead levels after 4 weeks without treatment with sub-chronic doses of ascorbic acid (98.2 ± 11 μg Pb/g egg-albumen) were not statistically different (*p*-value > 0.05) from those seen after 1 week of exposing hens to lead acetate. Interestingly, treatment with sub-chronic doses of ascorbic acid significantly (*p*-value < 0.01) decreased lead levels in egg-albumen samples to 62.5 ± 0.5 μg Pb/g egg-albumen). Lead levels in egg-albumen samples are shown in Fig. 3.

### Lead levels in egg-shell samples

**Fig. 4:**
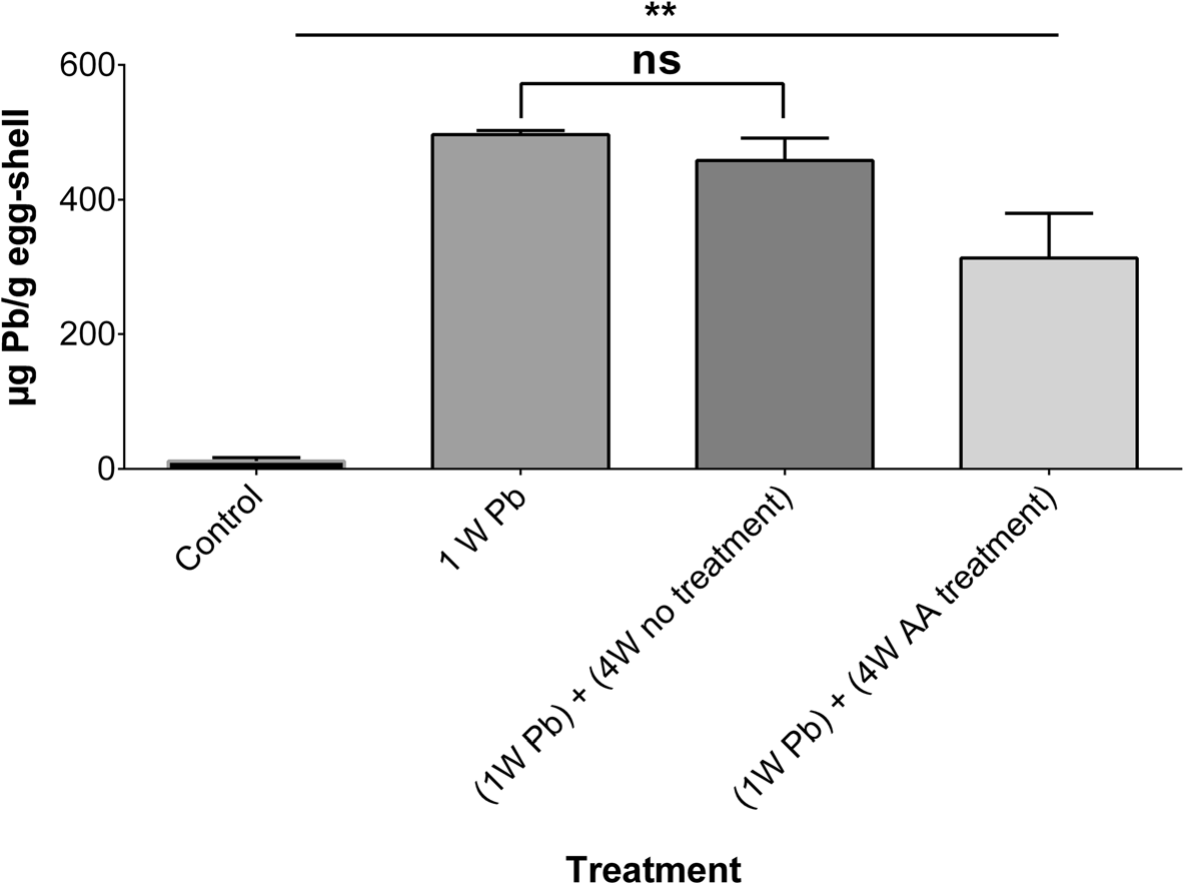
Lead (Pb) levels in egg shell samples at baseline (control), following exposure to lead acetate (200 mg/kg_body weight_/day) for 1 week (1W Pb), 4 weeks without (4W no treatment), and 4 weeks with (4W AA treatment) treatment with high daily doses (500 mg/kg_body weight_/day) of ascorbic acid. A total of 6 samples were analyzed. ns: not statistically significant, **: *p*-value < 0.01

The baseline lead levels were 11.5 ± 5.3 μg Pb/g egg-shell. Lead levels in egg-shell samples increased significantly (*p*-value < 0.01) to 496 ± 6.0 μg Pb/g egg-shell after exposing hens to lead acetate. Again, lead levels did not differ significantly (*p*-value > 0.05) after 4 week of no treatment with ascorbic acid (458 ± 33 μg Pb/g egg-shell) from those seen after 1 week of intentional exposure to the concentrated source of lead. Interestingly, lead levels were significantly (*p*-value < 0.01) reduced to 313 ± 67 μg Pb/g egg-shell after 4 weeks of treatment with sub-chronic doses of ascorbic acid. Lead levels in egg-shell samples are shown in Fig. 4.

### Comparison between lead levels in different hen egg parts

**Fig. 5:**
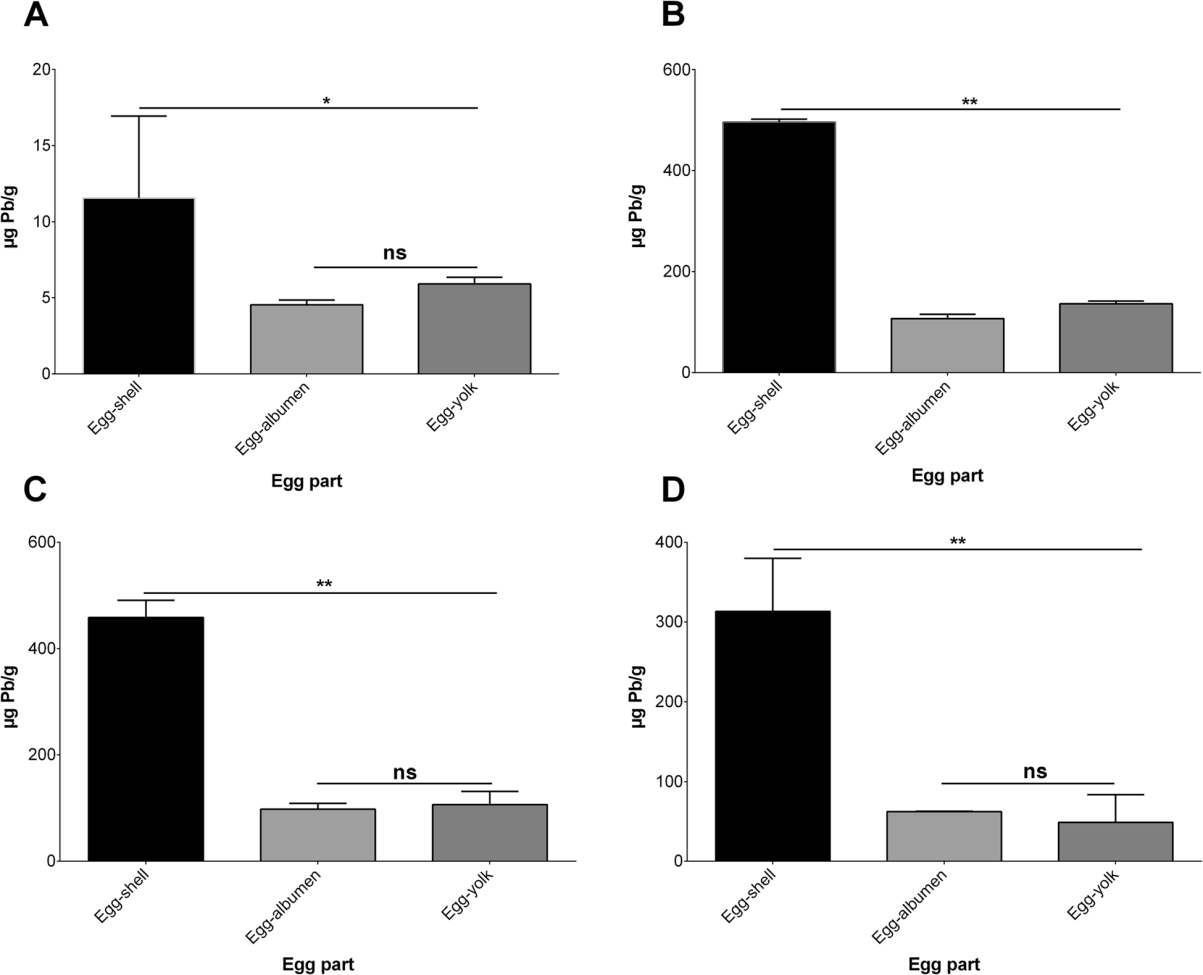
Comparison between lead levels at baseline (**a**), 1 week after intentional exposure of hens to 200 mg/kg_body weight_/day lead acetate for 1 week (**b**), 4 weeks without treatment (**c**), and 4 weeks of sub-chronic treatment with 500 mg/kg_body weight_/day ascorbic acid (**d**) in hen egg-shell, egg-albumen, and egg-yolk samples. ns: not statistically significant, **: *p*-value < 0.01, *: *p*-value < 0.05

At baseline, edible egg (albumen and yolk) samples contained comparable lead levels (Fig. 5a). After intentionally exposing hens to 200 mg/kg_body weight_/day lead acetate for 1 week, lead levels were significantly (*p*-value < 0.01) higher in egg-shell samples compared to egg-albumen and egg-yolk samples (Fig. 5b). Again, egg-yolk samples contained significantly (*p*-value < 0.01) higher lead levels than in egg-albumen samples. After 4 weeks of treatment or no treatment with ascorbic acid, egg-shell samples contained significantly (*p*-value < 0.01) higher lead levels than those quantified in egg-albumen and egg-yolk samples (Fig. 5c and d). Lead levels in egg-yolk and egg-albumen samples were not statistically different (*p*-value > 0.05). Comparison between lead levels in the different egg parts is shown in Fig. 5a-d.

## Discussion

This study reports statistically significant reduction in lead levels in various hen egg portions intentionally exposed to a concentrated source of lead followed by a sub-chronic treatment with ascorbic acid. Lead acetate levels were determined in eggshell, yolk and albumen samples separately before treatments (baseline), following 1 week ingestion of lead acetate, after 4 weeks of ascorbic acid treatment, and 4 weeks without ascorbic acid treatment.

Findings of this study are interesting because the group of mixed race hens used in the study are identical to those raised in the vicinities of homes of many raisers in the West Bank of Palestine [6, 29]. To the best of our knowledge, this study reports for the first time statistically significant reductions in lead levels in different hen egg parts following sub-chronic treatment with ascorbic acid. Findings from this study might help in bridging the gap of knowledge that existed in earlier experiments.

Findings of this study were consistent with those previously reported in the literature which showed that lead levels significantly increased in the blood and other parts of hen eggs following intentionally or environmentally exposing hens to a source of lead [1, 2, 6, 29, 30]. This study showed clearly elevated lead levels in egg shell, albumen, and yolk samples. When we sampled different hen egg portions during the acclimatization period and before exposing hens to the source of lead, egg shell, albumen, and yolk samples contained detectable amounts of lead at baseline. These findings might indicate the hens obtained from raisers were probably already environmentally exposed to lead. These findings are particularly interesting and consistent with those reported in different countries like Nigeria, India, and the US [1, 2, 30, 31, 39, 40]. Similarly, in a previous study conducted in Palestine, hen’s blood samples were shown to contain detectable lead levels at baseline and before intentional exposure [29]. Sampled water and feedstuff also contained detectable lead levels. Therefore, it would be interesting to sample hen eggs available commercially at retail stores and screen them for their heavy metals contents. Hen eggs contaminated with lead and other heavy metals could be an important source of human exposure, especially, when contaminated eggs are repeatedly consumed. The risk is multifold when children are exposed to lead. Current recommendations set a provisional tolerable intake in a week at 50 μg/kg of body weight for adults and 25 μg/kg of body weight for children [41].

Results of this study were consistent with those previously reported in which egg shell portions were shown to exhibit higher concentrations of lead after exposing hens to a source of lead [42]. Comparing lead levels in the edible part of hen eggs, in general, lead concentrations in albumen samples tended to be less than those in yolk samples. These results were consistent with the trends reported in a previous study [29]. Taken together, these findings suggest that hen egg yolk could be a considerable source of exposure to lead in adults and children who consume lead contaminated hen eggs.

In general, sub-chronic treatment with ascorbic acid was shown to reduce lead levels in different hen egg portions. This might suggest the possibility of supplementing hens’ feedstuffs with sources of ascorbic acid. The idea of supplementing feedstuffs with commercially available ascorbic acid might not be feasible due to economic reasons. Probably, supplementing feedstuffs with residues of citrus and other fruits and green leafy vegetables could be among alternative sources of ascorbic acid. The antioxidant capabilities of ascorbic acid are well-recognized. Many previous studies have shown abilities of ascorbic acid in scavenging free radicals. Previous studies have suggested that ascorbic acid might be able to bind to and remove lead [36, 43–45]. Additionally, ascorbic acid was also shown to alleviate the deleterious effects of exposure to lead like hepatotoxicity [46] and impairment of synaptic plasticity [43].

### Limitations of the study

The results of this study should cautiously be interpreted considering the following limitations. First, we did not sample and assess feedstuffs and water for lead contents. However, in previous studies, feedstuffs and water were shown to contain detectable concentrations of lead [6, 18]. This might explain baseline lead levels detected in different egg parts. Second, the number of eggs sampled and analyzed at each stage. In this study, 6 eggs were sampled and assessed for lead contents at each stage of the study. Sampling more eggs should have produced more reliable findings. However, 6 eggs were sampled at each stage of the study, each egg was separated into 3 components (shell, albumen, and yolk), and each part from each egg was analyzed separately. Third, in this study, treatment lasted for 4 weeks only. It would be interesting to follow up with the treatment for a longer period of time. Fourth, in this study we did not investigate if fruit residues that are supposed to contain dietary ascorbic acid could result in significant reduction of lead levels in hen eggs. Finally, it would have been a good idea to sample hen eggs commercially available on the market and assess their lead contents. This should have provided information on hen eggs as a source of exposure to lead, instead of intentionally exposing hens to a concentrated source of lead.

## Conclusions

In conclusion, our pilot study showed that sub-chronic treatment of hens with high doses of ascorbic acid could bring statistically significant reduction in lead levels after intentional exposure to a concentrated source of lead. Supplementing feedstuffs and water with sources of ascorbic acid could be beneficial in reducing lead levels in hen egg parts following environmental exposure. Further studies are needed to understand the mechanisms by which ascorbic acid accelerates the process by which lead is removed from the different egg parts.

## Data Availability

Data related to this study are either presented in the results section. Raw data can be obtained from the corresponding author on reasonable request.

## Abbreviations

ANOVA: Analysis of variance
G1: Group 1
G2: Group 2
LOD: Limit of detection
WHO: World Health Organization
